# Women’s experience of agency and respect in maternity care by type of insurance in California

**DOI:** 10.1371/journal.pone.0235262

**Published:** 2020-07-27

**Authors:** Eugene Declercq, Carol Sakala, Candice Belanoff

**Affiliations:** 1 Community Health Sciences, Boston University School of Public Health, Boston, MA, United States of America; 2 National Partnership for Women & Families, Washington, DC, United States of America; Florida State University College of Medicine, UNITED STATES

## Abstract

**Objective:**

Public insurance (Medicaid) covered 42% of all U.S. births in 2018. This paper describes and analyzes the self-reported experiences of women with Medicaid versus commercial insurance relating to autonomy, control and respectful treatment in maternity care.

**Methods:**

The sampling frame for the *Listening to Mothers in California* survey was drawn from 2016 California birth certificate files. The 30-minute survey had a 55% response rate. A secondary multivariable analysis of results from the survey included 2,318 women with commercial private insurance (1,087) or public (Medi-Cal) (1,231) coverage. Results were weighted and were representative of all births in 2016 in California. The multivariable analysis of variables related to maternal agency included engagement in decision making regarding interventions such as vaginal birth after cesarean and episiotomy, feeling pressured to have interventions and sense of fair treatment. We examined their relationship to insurance status adjusted for maternal age, race/ethnicity, education, nativity and attitude toward birth as well as type of prenatal provider, type of birth attendant and pregnancy complications.

**Results:**

Women with Medi-Cal had a demographic profile distinct from those with commercial insurance. In multivariable analysis, women with Medi-Cal reported less control over their maternity care experience than women with commercial insurance, including less choice of prenatal provider (AOR 1.61 95%C.I. 1.20, 2.17), or a vaginal birth after cesarean (AOR 2.93 95%C.I. 1.49, 5.73). Mothers on Medi-Cal were also less likely to be consulted before experiencing an episiotomy (AOR 0.30 95%C.I. 0.09, 0.94). They were more likely to report feeling pressure to have a primary cesarean (AOR 2.54 95%C.I. 1.55, 4.16) and less likely to be encouraged by staff to make their own decisions (AOR 0.63 95%C.I. 0.47, 0.85).

**Conclusions:**

Childbearing women with public insurance in California clearly and consistently reported less opportunity to choose their care than women with private insurance. These inequities are a call to action for increased accountability and quality improvement relating to care of the many childbearing women with Medicaid coverage.

## Introduction

The increasing concern with disrespect and abuse during pregnancy and childbirth [1] has led to calls to support greater maternal agency over the childbearing process, ([2] not simply as an ethical concern, but as a way to improve population health. The importance of maternity care to population health is coming into sharper focus with increasing understanding of the microbiome ([3], epigenetics [4], life course health development [5] and hormonal physiology.[6] Practices and quality variation within maternity care may favorably or adversely affect children’s long-term health. Similarly, the 85% of U.S. women who give birth one or more times ([7] experience both desirable and undesirable long-term health consequences related to maternity care. [8–10] The present maternal health crisis in the United States, with high rates of maternal mortality and severe maternal morbidity, and persistent extreme racial and ethnic inequities, [11] further underscores the priority of examining and improving policy and practice bearing on maternal health.

Medicaid, the nation’s medical assistance program for low-income individuals, covered 42% of all births in the United States in 2018 [12] and serves a disproportionately marginalized population. Because of its scope and population served, Medicaid can be a vehicle for public programs and policies to improve the care, experiences and outcomes of a large proportion of childbearing women and newborns. Medicaid also provides a safety net for at-risk populations, thus reducing the risks of adverse selection for private insurers [13] and Medicaid policies can have spillover effects on commercial insurance policies, [14] thus having even greater impact on the health of childbearing families.

One way to assess the impact of Medicaid is to compare childbearing women covered by Medicaid to those covered by commercial insurance. Given the major socioeconomic differences in the populations served by Medicaid and commercial insurance, adjustment for demographic characteristics is essential when comparing the care, experiences and outcomes of the two payer types. Even with adjustment, it is important to note that commercial insurance typically provides more generous reimbursement than Medicaid and could result in differences in care quality and experiences. A national analysis of all payments made for a woman and her baby from pregnancy through the newborn and postpartum periods found average commercial payments were twice those of Medicaid payments [15].

The National Quality Strategy of the Agency for Healthcare Research and Quality (AHRQ) highlights the importance of having individuals and families engaged in their care. [16] Engagement and patient activation have been associated with improvements in outcomes generally [17, 18] and specifically in maternal and infant health. [19] We examined whether indicators of maternal agency, a concept that encompasses a sense of women’s control and autonomy, [20, 21] differed by type of payer during prenatal, intrapartum and postpartum periods. Research comparing care of women with Medicaid and commercial coverage for maternity care generally reports on differences in use of interventions, with Medicaid beneficiaries less likely to experience cesarean birth overall, [22–25] or for specific diagnoses [26] or in one study experiencing no difference. [27] Mothers with Medicaid coverage have been found more likely to experience several indicators of support for vaginal birth after cesarean (VBAC). [28, 29] Other studies found less use of labor induction [22], epidural analgesia among vaginal births [30], and episiotomy [22] among women with Medicaid versus commercial insurance. Women using Medicaid were also more likely than those using commercial insurance to have a nurse-midwife as their birth attendant [31] and report being involved in shared decision making. [32]

The sole study we identified of childbearing women’s perceptions of experiences by payer type found women with Medicaid versus commercial coverage reporting lower levels of commitment to their primary physician and less trust in their primary physician and other physicians in the practice. [33] Women with Medicaid also were less likely to feel that they shared values with their main care provider; rated the quality of the service lower; were less satisfied with the quality of the care they received; were less likely to intend to return to the practice for future pregnancies and to refer other women to the practice; and felt less comfortable voicing a complaint. [33] This examination of mothers’ sense of influence over their environment and their experience of childbirth, by payer, is now 20 years old. The current study both updates and explores more broadly the relationship between payer type and women’s sense of control over their maternity experiences. Our research is a secondary analysis of results from *Listening to Mothers in California*, based on a representative sample of the nearly 500,000 women who gave birth in California hospitals in 2016.

While California residents give birth to one in eight infants in the nation, California has a distinctive maternity care environment. In this Medicaid expansion state, many women with Medi-Cal (the state’s Medicaid program) maternity coverage experience continuous coverage extending prior to their pregnancy and beyond the conventional period of postpartum coverage. [34] Pregnant women without insurance and with incomes at or below 200% of the federal poverty level are eligible for pregnancy-related Medi-Cal coverage. California women participate in Medi-Cal through both fee-for-service and managed care plans.

## Materials and methods

The *Listening to Mothers in California* survey was developed through a collaboration of investigators from the National Partnership for Women & Families, Boston University School of Public Health and the University of California, San Francisco (UCSF) Center for Health Equity, who worked with Quantum Market Research to administer the survey. The sampling frame for this study was drawn from California birth certificate files for births between September 1 and December 15, 2016. We excluded women less than 18 years of age, women with out-of-hospital births, women with non-singleton births, non-residents of California, women who could not participate in English or Spanish, and women who were not living with their baby at the time of survey participation. The recruitment of participants involved up to four invitation and reminder mailings, which included two inserts: invitation cover letters incorporating elements of informed consent and information cards on how to access the survey online via any device using a unique code that was provided. The card also indicated how to reach a telephone interviewer and learn more about the project. The survey was available in two languages, and 81% of the final sample participated in English and 19% in Spanish. We oversampled Black women, women with midwifery-attended births and those with a VBAC to have sufficient sample sizes to analyze the experiences of women within these smaller groups. The survey was conducted from February 22 through August 15, 2017. Sampled women were invited to participate on their own online using a smartphone or any other device, or with an interviewer via telephone. Respondents participated from 2 to 11 months after giving birth. Of those who completed the survey, 34% did so online, 28% did so by phone with an interviewer and 39% used both methods, typically starting on their own and finishing with an interviewer. [35] On average, the survey took a bit longer than 30 minutes to complete. The entire *Listening to Mothers in California* survey questionnaire and related materials are available at both nationalpartnership.org/LTMCA and chcf.org/listening-to-mothers-CA.

To better reflect a statewide profile of childbearing women aged 18 and older giving birth to single babies in California hospitals and account for nonresponses, UCSF analysts weighted the final sample using demographic and other variables from the 2016 Birth Statistical Master File to be representative of the full 2016 year of California births. Our final sample size of 2,539 represented a response rate of 55%. A detailed explanation of the methodology is presented in the *Listening to Mothers in California* report appendices. [35] The Committee for the Protection of Human Subjects of California’s Office of Statewide Health Planning and Development is the IRB of record and approved the study and subsequent protocol amendments. The UCSF IRB also approved the project. The California Department of Public Health Vital Statistics Advisory Committee approved access to birth certificate data. The data were fully anonymized before the authors received the analytic file. The analysis was completed using SAS (Cary, NC) version 9.4.

Given our focus on insurance coverage, we took extra steps to develop a valid measure of type of insurer. In California, in addition to the various options for Medi-Cal coverage, women may obtain maternity coverage through a marketplace of plans available due to the Affordable Care Act, through other government-supported programs and through employer-provided plans. While we couldn’t distinguish all sources of insurance, we worked with the California Department of Health Care Services to link to claims records in the Management Information System/Decision Support System (MIS/DSS) Warehouse and defined a Medi-Cal beneficiary as a respondent with a paid Medi-Cal claim for a 2016 birth in the MIS/DSS Warehouse. We defined commercially insured respondents as those without such a paid Medi-Cal claim who self-identified a commercial source of payment on the survey. Of the original 2,539 respondents, we identified 2,318 who were covered by either Medi-Cal or commercial insurance. Other women, including a very small number who reported no insurance, were excluded from present analyses. For one of our variables, our comparisons were limited to women who reported primarily speaking English in their homes. This was necessary because there was some uncertainty about the interpretation of the term “episiotomy” among respondents who did not primarily speak English in their homes.

For the multivariable analyses, we chose dependent variables that were significantly different by insurance payer in the bivariate comparisons and reflected women’s potential freedom of choice of care options, thereby representing elements of maternal choice and control. They were choice of prenatal care provider, ability to identify the type of physician providing prenatal care and attending birth, whether or not a woman with one or two prior cesareans was given a choice of having a VBAC and if so, if she was consulted about the decision. For a woman who had an episiotomy, we asked whether she had a choice in having it. In some cases, we chose a single measure (e.g., being mobile in labor) to represent a number of related variables (e.g., nonpharmacological pain relief measures). We also examined whether women gave birth in a supine position and if they reported feeling pressure from a health professional to have an epidural and/or a primary cesarean. We also examined whether or not a woman had a postpartum visit between 3 and 8 weeks after birth, and if so, was she asked about birth control and depression. Finally we assessed the relationship between insurer and whether a woman felt the maternity staff encouraged their own decision making and whether they felt they were treated unfairly because of their race/ethnicity, language spoken or insurance coverage. Since many of the analyses involved subgroups (e.g. choice of VBAC was only asked of women with a prior cesarean), Table 4 specifies the subgroup in each analysis.

We controlled for variables that might impact choice: type of either prenatal maternity provider or birth attendant (whichever was most relevant to the dependent variable), as well as race/ethnicity, maternal education, maternal age, whether or not there were any pregnancy complications reported on the birth certificate, and whether the participant had been born in the United States. To control for the effect of maternal attitudes, we included a variable measuring the agreement with the statement “childbirth is a process that should not be interfered with unless medically necessary.”

## Results

Among California women who had a birth in 2016, the prevalence of Medi-Cal coverage varied considerably across almost every demographic characteristic (Table 1). Medi-Cal coverage was more likely among women who were younger, Black or Latina, of higher parity, overweight or obese at the outset of pregnancy, born outside the United States, spoke Spanish in the home and had a high school or less education.

**Table 1:** Demographic characteristics of childbearing women covered by Medi-Cal and by commercial insurance.

Category (n = 2,318)	Medi-Cal		Commercial	
Mother’s Age	**%**	**95% C.I.**	**%**	**95% C.I.**
<25	77.3	73.0–81.1	19.3	15.7–23.3
25–29	54.3	50.1–58.3	42.2	38.2–46.3
30–34	33.0	29.5–36.6	62.0	58.2–65.6
35+	36.8	32.4–41.3	59.7	55.1–64.1
Mother’s Race/Ethnicity				
Non- Latina white	27.5	23.9–31.4	66.9	62.8–70.7
Non- Latina Black	54.0	46.8–61.1	40.6	33.7–47.8
Non- Latina Asian	23.7	19.2–29.0	69.8	64.1–74.9
Latina	69.1	66.2–71.8	28.8	26.1–31.6
Parity				
1	38.2	35.1–41.4	56.9	53.7–60.1
2	46.6	42.8–50.4	49.3	45.5–53.2
3+	68.8	64.9–72.5	28.5	25.0–32.4
BMI				
Underweight	35.5	30.2–41.2	57.6	51.7–63.3
Normal	42.3	38.9–45.7	53.9	50.5–57.3
Overweight	51.7	47.1–56.3	44.1	39.6–48.7
Obese	62.6	57.6–67.4	35.4	30.7–40.4
Marital Status				
Married	30.6	28.2–33.2	64.8	62.1–67.4
Living w/ someone	74.1	70.3–77.7	23.0	19.7–26.8
Single, never married	71.0	56.9–82.0	21.4	12.1–35.0
Birthplace				
US	44.2	41.6–46.8	52.1	49.4–54.7
Other country	56.9	53.2–60.5	38.6	35.1–42.2
Language at home				
English	36.8	34.1–39.5	59.2	56.4–61.8
Spanish	85.4	81.4–88.6	12.4	9.4–16.1
Asian language	26.6	19.8–34.6	61.1	52.5–69.1
Education				
High school or less	81.0	77.8–83.9	16.1	13.4–19.2
Some college	53.9	50.1–57.6	43.2	39.5–47.0
College	17.6	14.4–21.5	74.8	70.4–78.7
Some grad school+	9.8	7.1–13.4	85.4	81.3–88.8

a. Row totals do not equal 100% because of a small proportion of mothers who did not have either a Medi-Cal claim or report having commercial insurance.

Insurance coverage becomes the independent variable in subsequent analyses of the relationship of insurance status to maternal experiences, choices and respectful treatment (Tables 2–4). In the bivariate comparison of insurance status with provider choice and intrapartum experiences (Table 2), mothers with Medi-Cal coverage were twice as likely to report that they did not have a choice of provider for pregnancy and birth 25.6%, 95% C.I. (23.1%, 28.2%) compared to mothers with commercial insurance 12.8%, 95% C.I. (10.9%, 15.0%). They were also less likely than women with commercial insurance to have an obstetrician as their prenatal provider and much more likely 6.8%, 95% C.I. (5.4%, 8.5%) to 0.8%, 95% C.I. (0.4%, 1.5%) to report having a “doctor but I’m not sure which kind” as their prenatal provider. Women with Medi-Cal were more likely to report wanting a different kind of provider, generally preferring an obstetrician to the prenatal provider they had. In terms of birth attendant, women with Medi-Cal were almost three times as likely to not know what kind of doctor was attending their birth 19%, 95% C.I. (16.7%, 21.4%) compared to women with commercial insurance 6.4%, 95% C.I. (5.0%, 8.0%). There was no significant difference in the likelihood of using a doula. There was a substantial difference in attitude about birth based on level of support for the statement, “Childbirth is a process that should not be interfered with unless medically necessary.” A majority 56%, 95% C.I. (52.9%, 58.8%) of women with Medi-Cal strongly agreed with the statement, while only 39%, 95% C.I. (35.5%, 41.6%) of women with commercial insurance strongly agreed.

**Table 2:** Maternal experiences and attitudes toward birth, by type of payer.

	Insurer			
	Medi-Cal		Commercial	
**Provider Choice and Experience** (All women n = 2,318)	%	95% C.I.	%	95% C.I.
Had choice of provider*	73.4	70.8–75.9	87.1	84.9–89.0
Did not have choice*	25.6	23.1–28.2	12.8	10.9–15.0
Looked for cesarean rate of prospective birthing hospital*	29.5	26.9–32.3	35.5	32.5–38.5
Prenatal provider				
Obstetrician*	76.1	73.6–78.5	84.4	82.2–86.4
A doctor, not sure what kind*	6.8	5.4–8.5	0.8	0.4–1.5
Would have preferred a different kind of provider*	14.5	12.6–16.7	8.9	7.3–10.8
Of these, prefer a Midwife*	41.4	34.0–49.3	62.8	52.3–72.2
Birth Attendant				
Obstetrician*	67.9	65.2–70.6	77.9	75.6–80.1
A doctor, not sure what kind*	19.0	16.7–21.4	6.4	5.0–8.0
Midwife*	6.3	5.2–7.7	12.5	11.1–14.1
**Intrapartum Experiences** Vaginal births (n = 1,635)				
Attempted induction	41.2	37.8–44.7	47.0	43.4–50.7
Vaginal–pressure for induction	13.2	10.9–15.8	18.5	15.8–21.4
*VBAC issues* (Women with 1 or 2 prior cesareans = 423)				
Interested in VBAC	47.4	40.5–54.4	46.4	36.9–56.2
No option for VBAC*	65.4	58.5–71.7	38.9	29.8–48.9
*Discussed VBAC/repeat cesarean decision*				
Provider asked opinion*	64.9	56.6–72.4	87.5	79.6–92.7
Was woman’s decision	26.0	19.5–33.8	42.8	33.4–52.8
Was provider’s decision	27.4	20.6–35.5	13.5	8.1–21.8
**Medical interventions** (Vaginal births only n = 1,635)				
*Episiotomy* (English primary language) (n = 1,328)	15.8	12.1–20.3	17.1	14.0–20.7
*Given choice about episiotomy* (among those who experienced episiotomy, n = 141)*	10.4	4.7–21.2	31.3	22.2–42.3
*Pain relief medications* (Vaginal births n = 1,635)				
Epidural*	61.7	58.2–65.1	74.1	70.7–77.1
Woman felt pressured for epidural*	13.4	11.1–16.1	8.1	6.3–10.2
*Used no pain medication*	25.3	22.3–28.5	19.7	17.0–22.8
*Woman felt pressure to have a cesarean*	13.1	11.2–15.3	9.2	7.6–11.2
*Pregnancy complication reported on birth certificate*	19.2	17.0–21.6	18.6	16.3–21.2
*Mode of birth*				
Overall cesarean rate (All women n = 2,318)	33.6	30.8–36.4	28.1	25.4–31.0
Nulliparous term, singleton, vertex cesarean rate	28.8	24.0–34.2	24.4	20.5–28.7
Vaginal birth after cesarean (VBAC) rate (Women with 1 or 2 prior cesareans n = 423)	12.6	9.6–16.3	16.4	12.3–21.5
*Experiences of staff support* (% agree strongly)				
Staff encouraged maternal decision making*	46.9	43.6–50.2	54.3	50.9–57.6
Well supported by staff	73.6	70.6–76.4	76.7	73.8–79.5
Staff communicated well	76.4	73.5–79.0	72.3	69.1–75.2
*Strongly agree birth shouldn’t be interfered with unless medically necessary* (All women n = 2,318)*	55.9	52.9–58.8	38.5	35.5–41.6

Categories with an asterisk (*) indicate variables that differ significantly by payer in bivariate analyses (p < .05).

**Table 3:** Prevalence of postpartum experiences and perceived treatment, by payer.

	Insurer			
	Medi-Cal		Commercial	
**Immediate postpartum**	%	95% C.I.	%	95% C.I.
*Vaginal Births (n = 1*,*635)*				
Any skin-to-skin contact after birth?*	69.2	65.9–72.4	80.1	76.9–82.9
NICU admission*	12.7	10.5–15.2	6.5	4.9–8.5
*All women (n =* 2,318*)*				
Exclusive breastfeeding intent*	59.5	56.6–62.3	77.1	74.3–79.7
Staff strongly supported breastfeeding*	81.3	78.8–83.5	86.8	84.5–88.8
Exclusive breastfeeding at 6 months*	22.9	20.5–25.4	34.6	31.7–37.6
Breastfed as long as wanted to	43.7	39.0–48.6	41.0	35.5–46.8
**After Leaving Hospital**				
No postpartum visit*	12.3	10.4–14.4	5.7	4.4–7.4
Didn’t have postpartum visit due to insurance	9.4	5.3–16.2	2.7	0.4–16.7
Postpartum visit content				
Asked whether needs help for birth	83.6	81.0–85.9	91.7	89.5–93.4
Control*				
Asked about depression*	74.8	71.9–77.5	82.0	79.3–84.5
**Reported Treatment during hospital stay**				
Treated unfairly due to race/ethnicity*	6.5	5.2–8.1	2.3	1.5–3.4
Hispanic*	6.0	4.6–8.0	1.4	0.5–3.4
Non-Hispanic white	1.5	0.5–4.8	0.3	0.0–1.8
Non-Hispanic Black	8.7	4.5–16.1	13.8	6.8–25.8
Asian and Pacific Islander*	19.3	11.4–30.6	5.6	3.1–10.0
Treated unfairly due to language spoken*	7.4	6.0–9.1	1.7	1.1–2.7
English speakers*	3.8	2.4–5.9	1.1	0.6–2.3
Spanish speakers	11.5	8.5–15.4	5.8	1.8–17.3
Treated unfairly due to type of health	6.5	5.2–8.1	2.3	1.5–3.4
Insurance*				
English speakers*	9.0	6.8–12.0	0.7	0.3–1.6
Staff used harsh language	7.3	5.9–9.1	8.1	6.5–10.0
Staff handled roughly	7.8	6.3–9.5	8.3	6.7–10.2

Categories with an asterisk (*) indicate variables that differ significantly by payer in bivariate analyses (p < .05).

**Table 4:** Unadjusted and adjusted^a^ odds ratios for outcomes associated with Medi-Cal insurance status.

		Medi-Cal vs Commercially Insured (ref)			
	Population	Un-adjusted Odds Ratio	95% Conf. Interval	Adjusted^a^ Odds Ratio	95% Conf. Interval
**Prenatal**					
Did not have choice of prenatal provider ^b^	All women	2.36	1.88–2.96	1.61	1.20–2.17
Main prenatal care provider was doctor, but not sure what kind ^b^	All women	9.72	4.65–20.29	4.99	1.93–12.90
**Intrapartum**					
Birth attendant was doctor, but not sure what kind ^c^	All women	3.49	2.60–4.69	1.87	1.28–2.72
Not given choice for VBAC^b^	Women w/ 1 or 2 prior cesareans	2.96	1.79–4.92	2.93	1.49–5.76
If discussed VBAC option, provider asked mother’s opinion. ^b^	Women w/ 1 or 2 prior CS who discussed VBAC	0.26	0.13–0.53	0.32	0.12–0.84
Given choice about having episiotomy^c^	Had an episiotomy	0.25	0.10–0.67	0.30	0.09–0.94
Reported pressure for primary cesarean ^c^	Women with no prior cesarean	1.73	1.21–2.48	2.54	1.55–4.16
**Treatment**					
Staff encouraged woman to make decisions ^c^	All women	0.62	0.49–0.77	0.67	0.50–0.91
Felt treated unfairly due to type of insurance status ^c^	All women	11.40	5.66–22.96	12.74	4.99–32.52
Felt treated unfairly due to race/ethnicity ^c^	All women	2.96	1.82–4.83	2.84	1.44–5.59
Felt treated unfairly due to language spoken ^c^^,^ ^d^	All women	4.55	2.67–7.75	3.08	1.48–6.45
**Postpartum**					
Had a postpartum visit ^b^	All women	0.43	0.31–0.61	0.48	0.30–0.76
During postpartum visit, provider asked about birth control ^b^	Women with postpartum visit	0.47	0.35–0.63	0.44	0.30–0.64
During postpartum visit, provider asked about depression ^b^	Women with postpartum visit	0.65	0.52–0.82	0.71	0.52–0.97

a. Adjusted for maternal age (18–24, 25–29, 30–34, 35+), prenatal provider or birth attendant (midwife, obstetrician, other), race/ethnicity (Latina, Black not Latina, white not Latina, Asian/Pacific Islander not Latina), maternal education (less than college vs college or more), US born (no/yes), pregnancy complication (no/yes), and agreement with statement “childbirth shouldn’t be interfered with unless medically necessary” (strongly agree vs all other)

b. Prenatal provider used for “provider” in model

c. Birth attendant used for “provider” in model

d. Language spoken included in model and race/ethnicity was not.

Among women with a prior cesarean birth, there was no difference by insurance status in the level of interest in having a VBAC, but women with Medi-Cal were significantly more likely, 65.4%, 95% C.I. (58.5%, 71.7%) than women with commercial insurance, 38.9% 95% C.I. (29.8%, 48.9%) to report they did not have an option for a VBAC.

Among women with vaginal births, the reported rates of episiotomy were not different, however, women with commercial insurance were three times more likely 31.3%, 95% C.I. (22.2%, 42.3%) than those on Medi-Cal 10.4%, 95% C.I. (4.7%, 21.2%) to report being given a choice of whether or not to have an episiotomy. While women with Medi-Cal were less likely to report having an epidural than those with commercial insurance, they were more likely to report pressure from their providers to have an epidural. There was no difference in the reported rate of pregnancy complications on the birth certificate, but there was a non-significant trend toward a higher overall cesarean rate for women with Medi-Cal compared to those with commercial insurance.

In bivariate analyses of women’s experiences in the postpartum period (Table 3), women with Medi-Cal were twice as likely 12.3%, 95% C.I. (10.4%, 14.4%) as women with commercial insurance 5.7%, 95% C.I. (4.4%, 7.4%) to report not having a postpartum visit in the eight weeks following birth. Women with commercial insurance were more likely to report an intention to breastfeed, staff support for breastfeeding and to be breastfeeding at 6 months. Among those with a postpartum visit, women with commercial insurance were also more likely to report being asked about the need for birth control and whether they were feeling depressed at a postpartum visit. While reports of unfair treatment in the hospital were relatively rare overall, women with Medi-Cal were twice as likely to report that they were treated unfairly because of their race/ethnicity, particularly Asian and Pacific Islander women; four times as likely because of the language they spoke; and almost three times as likely because of their health insurance status.

In the multivariable analysis, we controlled for factors that might impact maternal agency and that varied significantly by insurance status in the bivariate comparison. Results from the adjusted models were largely consistent with the bivariate results, with only differences in reported pressure for an epidural not remaining significantly different after adjustment. Women with Medi-Cal coverage were 61% more likely than those with commercial insurance to report not having a choice of prenatal care provider. They were almost three times more likely aOR = 2.93, 95% C.I. (1.49, 5.76) to be told they could not have a VBAC. If they had a discussion with their prenatal provider about a VBAC, they were a third as likely to be asked their own opinion about the planned mode of birth. Among vaginal births, women with Medi-Cal were less likely to report being encouraged by staff to make their own decisions, while more likely to give birth in a supine position. More than twice as many women with Medi-Cal, aOR = 2.54, 95% C.I. (1.55, 4.16), reported feeling pressure to have a primary cesarean than those with commercial insurance. In the postpartum period they were half as likely to have a postpartum visit and, when they did, significantly less likely to be asked whether they felt depressed or needed help with birth control. While the absolute proportions reporting being treated unfairly were small, women with Medi-Cal were far more likely to report being treated unfairly because of their race/ethnicity, aOR = 2.84, 95% C.I. (1.44, 5.59), language spoken, aOR = 3.08, 95% C.I. (1.48, 6.45) and, especially, their insurance status, aOR = 12.74, 95% C.I. (4.99, 32.52).

## Discussion

With 42% of childbearing women in the United States insured by Medicaid, the impact of the program on population health is profound and represents a tremendous opportunity to influence maternal and newborn care, outcomes, experience and resource use. While a number of studies have examined maternal care processes by type of insurance, [22, 26–28, 36, 37] less attention has been given to the relationship between type of insurance coverage and maternal agency and respectful treatment in childbearing. [33] We used a population-based survey of 2,318 California women who gave birth in hospitals in 2016 to explore whether having commercial or public (Medi-Cal) insurance was related to their reports of respectful maternity care and their level of involvement in their maternity care and childbirth decision making. We found the demographics of women with commercial and public insurance differed, and women with public insurance were more likely to favor less medical intervention in their birth. In the adjusted analyses, women with public insurance were less likely than women with commercial insurance to report having a choice of their prenatal provider; knowing the type of doctor who was their primary prenatal provider and their birth attendant; among those with one or two prior cesareans, be asked about their preference for a VBAC and have the option of a VBAC; or, among those with an episiotomy, given a choice about whether to have this procedure. Women with Medi-Cal were less likely to report hospital staff encouraged them to make their own decisions, and more likely to report feeling pressured to have a primary cesarean. A sense of being treated unfairly because of their race/ethnicity and the language they spoke was significantly more likely among women on Medi-Cal than those on commercial insurance. Women using Medi-Cal were also much more likely to report discrimination on the basis of insurance status, confirming earlier research. [38] In the postpartum period, they were less likely to have a postpartum visit and, when they did, to be asked by their postpartum providers about key issues like birth control or feelings of depression.

Notably, in the overall *Listening to Mothers in California* survey, women with Medi-Cal expressed a high level of interest, comparable to those with commercial insurance, in forms of care that typically involve greater choice, more personalization, fewer interventions and high levels of satisfaction, should they give birth in the future. For example, whereas just 6% had a midwife birth attendant for their 2016 birth, 53% had an interest in a midwife for a future birth and 55% had an interest in a doula, figures comparable to women with commercial insurance. [35] Greater access to such high-value forms of care would be concordant with expressed wishes of many Medi-Cal beneficiaries, could lead to better birth outcomes and experiences and could reduce substantial maternity-associated costs for taxpayers. Women with Medi-Cal were also more likely than women with commercial insurance to agree that childbirth should not be interfered with unless medically necessary, and we used this attitudinal variable as a covariate in making adjusted comparisons.

This paper is subject to several limitations. We caution about generalizability to the U.S. as a whole or differences between public and private insurance to other nations. While this survey is representative of California women who gave birth in 2016, compared with the national population of childbearing women, greater proportions in California were born out of the country (primarily in Mexico), are Spanish speakers and identify as Latina or as Asian and Pacific Islander, and smaller proportions identify as white and Black. Hence, California is in fundamental ways not a microcosm of the U.S. There is also the possibility that, despite a wide array of confounders included in our models, remaining confounding variables are not accounted for. For example, we did not have institutional measures concerning levels of reimbursement, hospital maternity care culture, [39, 40] quality of care or values of providers, and hospital patient mix, all of which may influence the treatment of women with Medicaid. [41] Nonetheless this paper represents one of the largest studies to date of insurance coverage and maternally-reported experiences in childbirth and, to our knowledge, the only one explicitly examining the association of maternal choice, autonomy and respect with insurance status.

The picture that emerges for women with Medi-Cal in California suggests the work of advocates who have long sought to increase women’s experience of respectful care and control over their childbirth experiences is not over. [42] While improvements that were made in the past several decades have likely resulted in greater maternal agency, we found they were more likely to have reached women with commercial rather than public insurance. These findings suggest an experience for women with Medi-Cal coverage akin to an earlier time when women were expected to be more passive participants in birth. [43] Our results identify a major opportunity to improve the care of the more than 200,000 births (more than 5% of all births in the U.S.) annually in California. Given the reach of Medicaid nationally, combined with its influence on commercial insurance coverage, the opportunity to improve care may apply more broadly in the U.S. While opportunities to foster agency, autonomy and respect are greatest for women covered by Medicaid, our data also identified opportunities to improve care provided to women with commercial insurance as well. Overall, our results point to significant opportunities to improve the quality, experiences, outcomes and expenditures on behalf of childbearing women and newborns at a time when our nation is struggling with a maternal health crisis and persistent, egregious disparities.

## Supporting information

S1 Table Question formats for dependent variables. (DOCX)
