# Supplementary material for: Women’s experience of agency and respect in maternity care by type of insurance in California
Source: PLoS One. 2020 Jul 27;15(7):e0235262. doi: 10.1371/journal.pone.0235262 (PMC7384608; doi:10.1371/journal.pone.0235262)
Supplement: S1 Table — (DOCX) [file pone.0235262.s001.docx]

**S1 Table**

**Question formats for dependent variables.**

| **Item** | **Question** | **Responses – Bold indicates positive for agency** |
| --- | --- | --- |
| Did not have choice of prenatal provider | *Did you have a choice about which maternity care provider you had for your pregnancy care (prenatal care)?* | **Yes, I had a choice**  No, I had no choice; my maternity care provider was assigned to me |
| Main prenatal care provider was doctor, but not sure what kind | *Which type of maternity care provider most often provided your care during pregnancy?* | - **An obstetrician-gynecologist doctor (could be called OB or ob-gyn)** - **A family medicine doctor** - A doctor but I’m not sure what type - **A midwife (could be called CNM)** - **A nurse practitioner (NP) or other nurse who is not a midwife** - A physician assistant (PA) |
| Birth attendant was doctor, but not sure what kind | *Which type of maternity care provider delivered your baby on [date]?* | - **An obstetrician-gynecologist doctor (could be called OB or ob-gyn)** - **A family medicine doctor** - A doctor but I’m not sure what type - **A midwife (could be called CNM)** - **A nurse practitioner (NP) or other nurse who is not a midwife** - A physician assistant (PA) |
| Not given choice for VBAC | *In your recent birth, did you have the option of planning a vaginal birth after having had a previous c-section (VBAC)?* | **Yes**  No |
| If discussed VBAC option, provider asked mother’s opinion. | *Did your maternity care provider ask whether* ***you*** *wanted to schedule a c-section?* | **Yes**  No |
| Given choice about having episiotomy | *Did you have a choice about whether to have an episiotomy?* | **Yes**  No |
| Reported pressure for epidural | *Did you feel pressure from any health professional to use epidural for pain relief?* | Yes  **No** |
| Reported pressure for primary cesarean | *Did you feel pressure from any health professional to have a c-section?* | Yes  **No** |
| Had a postpartum visit | *Between the time you left the hospital after birth and 8 weeks after the birth, how many* ***office visits*** *did you have with a* ***maternity care provider for yourself?*** | 0  **1+** |
| During postpartum visit, provider asked about birth control | *During your postpartum office [visit/visits] in the first 8 weeks after birth, did any maternity care provider ask if you needed help with a method of birth control?* | **Yes**  No |
| During postpartum visit, provider asked about depression | *During your postpartum office [visit/visits] in the first 8 weeks after birth, did any maternity care provider ask if you were feeling depressed?* | **Yes**  No |
| Staff encouraged woman to make decisions | *How much do you agree with the following statements about your recent experience of labor and birth? The delivery room staff encouraged me to make decisions about how I wanted my birth to progress.* | **Agree strongly**  **Agree somewhat**  Neither agree nor disagree  Disagree somewhat  Disagree strongly |
| Felt treated unfairly due to type of insurance status | *During your recent hospital stay when you had your baby, how often were you treated unfairly because of the type of health insurance you had or because you didn’t have health insurance?* | **Never**  Sometimes  Usually  Always |
| Felt treated unfairly due to race/ ethnicity | *During your recent hospital stay when you had your baby, how often were you treated unfairly because of your race or ethnicity?* | **Never**  Sometimes  Usually  Always |
| Felt treated unfairly due to language spoken | *During your recent hospital stay when you had your baby, how often were you treated unfairly because of the language you spoke?* | **Never**  Sometimes  Usually  Always |
